# Supplementary material for: Umbilical cord length and neurodevelopmental disorders, a national cohort study
Source: PLoS One. 2025 Apr 23;20(4):e0322444. doi: 10.1371/journal.pone.0322444 (PMC12017576; doi:10.1371/journal.pone.0322444)
Supplement: S1 Supplement — (DOCX) [file pone.0322444.s001.docx]

**Supplement**

**Neurodevelopmental disorders ICD-9 and ICD-10 Codes**

ICD 9 ICD10

Cerebral palsy 343 G80

Intellectual disability 317-319 F70-F79

ADHD 314 F90

ASD (Autism spectrum) 299 F84

Epilepsy 345 G40-G41

Impaired vision 369 H54

Impaired hearing 389 H90-H91
