# Supplementary material for: Umbilical cord length and neurodevelopmental disorders, a national cohort study
Source: PLoS One. 2025 Apr 23;20(4):e0322444. doi: 10.1371/journal.pone.0322444 (PMC12017576; doi:10.1371/journal.pone.0322444)
Supplement: Supplementary file 2 [file pone.0322444.s002.docx]

**Outcome**	**Exposure** Umbilical cord length	**Odds ratio** (95% credible interval)*	**Odds ratio** (95% credible [illegible]
ADHD	Long cord	1.14 (1.09-1.20)	1.07 [illegible]
ASD	Long cord	1.13 (1.03-1.22)	1.12 [illegible]
ASD	Short cord	1.41 (1.06-1.80)	1.41 [illegible]
ID	Short cord	2.29 (2.08-2.52)	1.45 [illegible]
Impaired hearing	Short cord	1.38 (1.27-1.50)	1.16 [illegible]
Epilepsy	Short cord	1.30 (1.17-1.44)	1.14 [illegible]
CP	Long cord	1.34 (1.13-1.57)	1.15 [illegible]
CP	Short cord	1.30 (1.07-1.59)	1.14 [illegible]

*Bayesian regression, unadjusted. **Bayesian regression, adjusted for simulated strong confounding

Følsomhet Intern (gul)
