# Supplementary material for: Umbilical cord length and neurodevelopmental disorders, a national cohort study
Source: PLoS One. 2025 Apr 23;20(4):e0322444. doi: 10.1371/journal.pone.0322444 (PMC12017576; doi:10.1371/journal.pone.0322444)
Supplement: Attachment — Submitted filename: Response to reviewers_PONE-D-24-37689_final_25.02..docx [file pone.0322444.s005.docx]

Response to review

**PONE-D-24-37689**

Dear Academic Editor Shivanand Kattimani

We thank you and referees for useful comments. Please find our responses to the comments itemised below.

3. Thank you for stating the following financial disclosure:

 [The project was supported by the Gerda Meyer Nyquist Gulbrandson and Gerdt Meyer Nyquist’s Fund, and the Norwegian SIDS and Stillbirth Society [https://lub.no/](https://eur03.safelinks.protection.outlook.com/?url=https%3A%2F%2Flub.no%2F&data=05%7C02%7Ccathrine.ebbing%40helse-bergen.no%7Cb0148c0f78734297548708dd4b98b0bd%7Cbdcbe535f3cf49f58a6afb6d98dc7837%7C0%7C0%7C638749841809947545%7CUnknown%7CTWFpbGZsb3d8eyJFbXB0eU1hcGkiOnRydWUsIlYiOiIwLjAuMDAwMCIsIlAiOiJXaW4zMiIsIkFOIjoiTWFpbCIsIldUIjoyfQ%3D%3D%7C0%7C%7C%7C&sdata=m3RZYEesVW9VYo3xIsklRRxfH4uoQG3AMwwzRRdfx6A%3D&reserved=0). Prof. Dag Moster was awarded the grants. The funders played no role in conducting the research and writing the paper].

Please state what role the funders took in the study.  If the funders had no role, please state: "The funders had no role in study design, data collection and analysis, decision to publish, or preparation of the manuscript."

- ***Reply:*** *this is now changed.: Should read:*

*“The project was supported by the Gerda Meyer Nyquist Gulbrandson and Gerdt Meyer Nyquist’s Fund, and the Norwegian SIDS and Stillbirth Society* [*https://lub.no/*](https://eur03.safelinks.protection.outlook.com/?url=https%3A%2F%2Flub.no%2F&data=05%7C02%7Ccathrine.ebbing%40helse-bergen.no%7Cb0148c0f78734297548708dd4b98b0bd%7Cbdcbe535f3cf49f58a6afb6d98dc7837%7C0%7C0%7C638749841809947545%7CUnknown%7CTWFpbGZsb3d8eyJFbXB0eU1hcGkiOnRydWUsIlYiOiIwLjAuMDAwMCIsIlAiOiJXaW4zMiIsIkFOIjoiTWFpbCIsIldUIjoyfQ%3D%3D%7C0%7C%7C%7C&sdata=m3RZYEesVW9VYo3xIsklRRxfH4uoQG3AMwwzRRdfx6A%3D&reserved=0)*. Prof. Dag Moster was awarded the grant with grant number 102230106. The funders had no role in study design, data collection and analysis, decision to publish, or preparation of the manuscript.”*

…we will change the online submission form on your behalf.

- *Thank you.*

**Reviewer: 1**

 The objectives of this paper has been discussed and explored as mentioned. Proper ethical clearance has been taken from concerned departments. Strength and limitations have been mentioned.

However in the methodology there is no clear definition of what is long and what is short cord. 108 "Exposures were short below 5th percentile and long above 95th percentile". This needs to have a reference to gestational age specific umbilical cord growth chart, as data regarding term and preterm and association with umbilical cord length and NDD have been presented in your study.

- ***Reply:*** *We thank you for this comment****.*** *The percentiles of cord length were short (<5^th^ empirical percentile) or long (≥95^th^ empirical percentile) based on the cord lengths in the total study population. Empirical percentiles were constructed, based on gestational age at birth.*

*We have previously published reference ranges for cord length in PLOS one (reference no 12 in the current manuscript) ^1^. However, as the current study population is the same as in Linde et al 2018, we calculated and applied empirical percentiles based on the current study population. The method applied is well described in the reference paper (Linde, 2018). We have amended the text to make this clearer:* The paragraph now reads:
*“Exposures were short (<5^th^ percentile) and long (≥95^th^ percentile) umbilical cord. The cord length percentiles were empirical, i.e. constructed based on the distribution of umbilical cord length by parity, sex and gestational age in the study population. Since cord length increases with fetal size, we additionally constructed birthweight-specific percentiles for cord length. All data in the MBRN are registered prospectively. The attending midwife or physician conducted the examinations of the afterbirth and the neonate and entered the requested information into the registration form shortly after delivery. All neonates were examined by a physician and any malformation was noted. The umbilical cord was measured with a disposable tape and length reported in centimeters.”*

In the Covariates, maternal socioeconomic status has not been considered and discussed, though Figure 1 acyclic graph has included sociodemographic as a covariate.

- ***Reply*:** *Thank you. We had access to important socioeconomic variables, described in the “****Covariates****” section; ”maternal and paternal age and educational level, …marital status of the mother… maternal and paternal level of education (categorized into five levels) and immigrant status…”. We have added a footnote to Figure 1 Directed acyclic graph: “Social demographic factors contain maternal and paternal education level, marital status of the mother, immigration status of the child.”*

  There was brief mention of the mothers' BMI and Ethnicity association with umbilical cord length. 205 "we repeated the analysis with NDD outcome restricted to cases with information on maternal BMI. We found no effect of maternal BMI on the risk estimate. This statement needs to be supported by data.
- ***Reply:*** *We carried out analyses in the selection of the population where BMI was available. Including BMI in the model did not change the results; for the association between a long cord and ADHD OR in this subset was 1.07 (95%CI 0.93-1.24) and including BMI in the model aOR 1.02 (95%CI 0.88-1.17) and short cord and ID; OR 2.67 (95%CI 2.06-3.47) including BMI in the model; aOR 2.75 (95%CI 2,12-3.58). Please see the results in the table below. At the discretion of the Editor, we may add this as supplemental material, although we feel this may be too much information. The paragraph now reads:*

*“Since maternal BMI before pregnancy was available only in a subset of the population , we repeated the analyses (with NDD as outcomes) restricted to cases with information on maternal BMI (n=174,692). We found no effect of adjusting for maternal BMI on the risk estimates. Likewise, no effect was observed on the risk estimates when we adjusted for ART.”*

| Population with 174692 BMI data: | | |  |  |  |  |  |
| --- | --- | --- | --- | --- | --- | --- | --- |
| **Exposure** | **Outcome** | **OR** | **95%CI** |  | **aOR with BMI** | **95%CI** |  |
| long cord | ADHD | **1.07** | 0.93 | 1.24 | **1.02** | 0.88 | 1.17 |
| short cord | ID | **2.67** | 2.06 | 3.47 | **2.75** | 2.12 | 3.58 |
| short cord | Impaired hearing | **1.42** | 1.17 | 1.74 | **1.45** | 1.19 | 1.77 |
| short cord | Epilepsy | **1.48** | 1.16 | 1.89 | **1.51** | 1.18 | 1.92 |
| short cord | CP | **1.47** | 0.91 | 2.37 | **1.52** | 0.94 | 2.44 |
| long cord | CP | **1.18** | 0.75 | 1.86 | **1.12** | 0.71 | 1.76 |

Figure 4 of data sheet needs to be clearly labelled

- ***Reply:*** *We have now inserted the requested information in the Figure text.*

*Figure text now reads:*

**“Figure 1 Directed acyclic graph (DAG) describing the relationship between pregnancy, maternal and paternal factors, umbilical cord length and neurodevelopmental disorder in the child. White nodules represent covariates that are included in the model.** Social demographic factors contain maternal and paternal education level, marital status of the mother, immigration status of the child. **Grey nodule; Unknown confounding factors, e.g. genetic confounding.**

**Figure 2 The prevalence of neurodevelopmental disorders according to year of birth in Norway**

**Figure 3 The prevalence of neurodevelopmental disorders according to cord length percentile**

**Figure 4 Long or short umbilical cord (≥95^th^ or <5^th^ percentile) and risk of neurodevelopmental disorders among singletons. Panel A; exposure Long Cord, panel B; exposure Short Cord.**

**CP; Cerebral Palsy, ADHD;** Attention-deficit/hyperactivity disorder, ASD; Autism Spectrum Disorder**; ID; Intellectual Disability, OR; Odds Ratio, CI; Confidence Interval”**

Reviewer #2: Very important study.

- ***Reply:*** *Thank you.*
- calculation of sample size need clarification in methodology.
- ***Reply:*** *This is an observational population study, including all births in Norway during the study period, thus a sample size calculation is not relevant in this setting.*

Definition of short and long umbilical cord criteria or guideline and references is not addressed well in methodology.

- ***Reply:*** *Thank you! Please see reply to Reviewer no1 above.*

Avoid the abbreviations on abstract.

- ***Reply;*** *this is difficult due to word limit. The guide for authors states that abbreviations should be avoided ”if possible”. In our situation both ADHD and ASD should be easier to read than the full description.*

The control of variances or standard competence process of midwife or physician who examine the neonate and measure the umbilical cord is not clear.

- ***Reply:*** *We have included a reference in the discussion section regarding the strengths and limitations (ref no 53 in the manuscript ^2^). This study was a validation study of the classification and measurements of the placenta, membranes and cord carried out by midwives in a clinical setting. The inter- and intra-observer study showed no significant differences regarding placental weight and cord length, indicating that the validity of data regarding placenta and cord in the Norwegian birth register is sufficiently high to justify* *future large-scale epidemiologic research based on this database.*

1.	Linde LE, Rasmussen S, Kessler J, Ebbing C. Extreme umbilical cord lengths, cord knot and entanglement: Risk factors and risk of adverse outcomes, a population-based study. *PloS one*. 2018;13(3):e0194814. doi:10.1371/journal.pone.0194814

2.	Sunde ID, Vekseth C, Rasmussen S, Mahjoob E, Collett K, Ebbing C. Placenta, cord and membranes: a dual center validation study of midwives' classifications and notifications to the Medical Birth Registry of Norway. *Acta Obstet Gynecol Scand*. Sep 2017;96(9):1120-1127. doi:10.1111/aogs.13164
